# New Drugs, Appliances, and Things Medical

**Published:** 1890-03-15

**Authors:** 


### NEW DRUGS, APPLIANCES, AND THINGS MEDICAL.

[All preparations, appliances, novelties, etc., of which a notice is desired, should be sent to The Editor, The Lodge, Porchester Square, W.]

### "THE CREOLIN FORMULARY."

We have received from Mr. Helbing, F.C.S., a copy of the above, which he has compiled with great care. It is a most useful little work containing formulæ for the use of creolin in every shape in which that most valuable antiseptic has been used. The headings run through the whole alphabet from abortion to wound treatment. In addition, the name of the author from whom the prescription is taken is given. As creolin is now so largely used for antiseptic and disinfectant purposes, and seems to be practically a safe preparation, the issuing of the above compendium will fill a want. We may add that the author publishes the book at 63, Queen Victoria Street, E.C.

### PENNY QUININE.

The above is the name given to the latest form of cheap quinine selling which is before the public. Mr. Rivers Hicks, of Savage Gardens, Tower Hill, has put on the market penny boxes containing four grains of sulphate of quinine, either in the form of one pill for fever attacks, two two-grain pills for children's febrile conditions, or four one-grain pills for ordinary tonic purposes. He states in his prospectus that it is for the poorer class of the world's inhabitants that he has made this venture. We think that in aguish districts and in India and the Colonies the idea may prove useful. The pills are well made, and we have tested them and find them to contain good sulphate of quinine. It is owing to the unprecedentedly low price of the drug that this attempt could prove a commercial success, though if it does the profits are likely to be fairly good. We are against the public dosing itself with drugs without skilled advice, but think that in the districts above mentioned this form of quinine administration may be allowed. One word about quinine. Many people have an idiosyncracy with regard to it, deafness, headache, and sometimes a rash being caused by its use. During the late epidemic it was widely stated that the ammoniated tincture of quinine was a prophylactic, and would prevent an attack. We saw several cases of overdosing, but not one authentic case of prevention of the onset of the disease. It was useful as a general tonic after the acute stage had passed.

### COLOURING MILK FOR SALE.

In answer to Mr. F. A. Bennett, who writes to ask our opinion as to the advisability of using a fluid (of which he sends us a sample) to colour milk before sale in order to improve its appearance, we beg to say that on no account ought anything to be added to milk by the producer or vendor. Mr. Bennett states that the agent for the fluid told him that eight out of ten vendors or cowkeepers used the fluid. All we can say is that if it were known, they ought to be officially warned of the illegal nature of the proceeding. The commonest substance used is annato, and we suspect the sample of fluid sent to us is a solution of this vegetable colouring matter. The fluid has a most disagreeable odour and far from pleasant taste, and in many of its reactions agrees with the descriptions of annato. Mr. Bennett wisely states that he would not on any account use the fluid if it were likely to be hurtful, especially to infants. We can inform him that there is on record a fatal case of poisoning. An infant by mistake was given a small dose of a strong solution of annato, and died shortly afterwards. Finally, we would state that the principle involved in selling a sophisticated article is distinctly an immoral one, besides involving the risk of fine or imprisonment.

### "CEREALINE FLAKES" OF THE CEREALINE MANUFACTURING COMPANY, COLUMBUS, INDIANA.

We have received samples of the above from Thurber, Gates, and Co., Eastcheap. After a careful examination, chemically and microscopically, we find them to be a most excellent form of manufactured maize flour. From the process [of] manufacture one would expect to get an easily digested form of carbo-hydrate food, and we think this has been obtained. The best maize, after preparation, is cracked into small fragments; these are steamed, and then passed through closely-set heated rolls and pressed into thin flakes. These are then packed and placed on the market. From the published analyses we find that cerealine flakes contain a good percentage of albuminoids and fats, and that the amount of carbo-hydrates is also large. It is claimed for them that they form a most economical basis for bakers' mush and for setting ferment, as well as for ordinary domestic purposes. The question as to their suitability for infants' food has been asked. As the starch granules have been well cracked by heat in the process of manufacture, and as there is a good amount of soluble starch and destrin present, we think that where farinaceous food is necessary for children that this, in conjunction with milk, will form a very valuable food. In conclusion, we may state that our cook speaks highly of the flakes, and, judging by the custard pudding and cheese soufflet she produced with them, we think her praises are well deserved. We may add that the firm send out a useful pamphlet of receipts with the packages of flakes.

### ASPINALL'S ENAMEL PAINTS.

Of all the silent revolutions of the world none has probably been more productive of good than that effected by the introduction of Aspinall's Enamel. Neither rank, position, nor means interpose any bar to the use of this article by thrifty housewives of all classes. Utility apart, it is probable that no modern invention has given more genuine or lasting pleasure than that which mistresses of households and ladies of all ranks have experienced in the use of this paint. One secret of its success has been the thought and care which has been expended in bringing the article to market in a useful form. We have received a set of samples of Aspinall's Enamel, and have been much struck with the care and ingenuity expended upon the air-tight tins, the attractive boxes containing the metallic paints, and the strength and good quality of the brushes. This care is further manifested in the directions for use; one of them requests that the bare hand may never rest upon the article being enamelled, and another that all articles may be well washed in warm water with soda in it before the enamel is applied. The variety and perfection of the colours of these paints is remarkable, and the artistic effects which may be brought about by their use in capable hands are unlimited. It would be interesting to know how many articles of old furniture of every description have been rendered smart and attractive at a trifling expense, to the no small pleasure and satisfaction of the owners. Aspinall's enamel and washable water paints have come as a boon and a blessing to women especially, and there are not a few of the sterner sex who cheerfully acknowledge their indebtedness to their manufacturer and inventor. A careful trial, extending over a lengthened period, has convinced us that they have become a necessity in every well-managed household, and that no thrifty housekeeper from henceforward will consider her storeroom complete without a supply of them. The manufacturers are fully justified in stating that any eyesore in a house can be made into an ornament, even by a child, in a very short space of time by applying Aspinall's enamel, as no special knowledge is required, and complete success is easy of attainment if the most ordinary care be used. Hospital matrons, sisters, and nurses will find these paints indispensable for purposes of decoration.

